# A Singular Case of Monomania

**Published:** 1852-07-01

**Authors:** 


### A SINGULAR CASE OF MONOMANIA.

A VERY curious affair is announced to come before the tribunals in a short time, wherein the medical faculty of Paris will be greatly interested. One of the numerous victims to the disease which, according to Broussais, makes more ravages in this one city than in the rest of Europe put together—the terrible, relentless, inexorable *idée fixe*—died at the Hospital Beaujon a short time ago, leaving a considerable sum behind him. For seventeen years he had never slept two nights following in the same lodging, nor during that time had he taken his meals at the same place; and in spite of the easy circumstances in which he had lived—in spite of the vigilance of kind friends and relations, the *idée fixe* has chased him to the hospital after all! Well might poor Broussais express such alarm at the *idée fixe*, the pious horror of which had at last become his own. According to Broussais, every man was under the influence of the *idée fixe*, and, by his first question to the patient, he was invariably determined as to the direction in which it lay. "Are you feverish?" would he say to the sick man who came to consult him. "Very," was most frequently the reply. "Restless, hey?—fidgetty at night, hey?—tormented and uneasy, hey?" To all this the patient would, of course, assent. "Then, monsieur, you have surely got some *idée fixe! Ah! prenez garde à l'idée fixe!*" It is a curious fact that, however strongly the supposition was denied at first, Broussais seldom failed to elicit something like an avowal of some pet anxiety or other, which by its nature he pretended to trace either to the brain, the stomach, or the bowels. Monsieur A——, the patient who has just died at Beaujon a martyr to the *idée fixe*, was formerly an *employé* in the Tuileries under Charles the Tenth, and held a post of high trust and confidence. In July, 1830, when the mob entered the palace, so great was the respect and consideration in which he was held, that his apartment was unvisited, and afforded shelter to many of the *gardes du corps*, who otherwise would have been sacrificed to the fury of the people. He was allowed to retire at his leisure from the place he had occupied so long without the slightest molestation, taking with him his goods and chattels, and not in any way experiencing any more annoyance than the removal from one house to another must always occasion. He went to live with his wife and family in the Faubourg St. Honoré, announcing his intention of retiring altogether from public life. His mind was perfectly tranquil as to the future, for he had been prudent in his expenses, and had saved a sufficient fortune to ensure perfect ease and comfort for the remainder of his days. No symptoms of madness had ever been exhibited by himself or any member of his family, nor had previous pre-occupation been observed in his manner, when suddenly, on the 14th of November following the July of the Revolution, while at dinner, he laid down his knife and fork, and, turning to his wife, exclaimed, with a face full of consternation, "I have just remembered that I have to go to Vierzon." "Why so?" returned his wife, perfectly astonished at the sudden announcement. "To present myself at the drawing of the conscription." His wife laughed heartily at the idea. He was past fifty years of age—long past the time for entering the army; and his threat of repairing to Vierzon to present himself to the mayor was considered by her as an unaccountable joke. She knew he had escaped in his youth by making a journey over the frontier at the time of drawing; she knew that he had always been subject to the inquisition of the police until liberated by his age from all obligation of the kind, and she dismissed the affair altogether from her mind, and saw him depart for his walk, "as was his custom of an afternoon," without the slightest feeling of uneasiness. Her surprise may therefore be imagined when, late that evening, she received a note from her husband, wherein he requested her not to sit up for him that night, as he should not return, being well aware that the police were on the look out for him, and that, if taken, he should be condemned to imprisonment for *contumace* as regarded the conscription! From that hour he wandered forth, finding no rest, seeking no company, pursued by the remorseless *idée fixe* which had taken possession of his soul; one night sleeping in the Faubourg St. Antoine, the next at the Barrière de l'Etoile; sometimes dining on the Boulevard, and passing the night at Vincennes, to rise before daylight in order to avoid the gendarmerie, and then hurrying straight to Passy to snatch a hasty meal, and hurry off to St. Germain. This life he has led for seventeen years, and at length, worn out with anxiety and fatigue, he was brought from a small lodging-house in the Rue de la Pepinière. At the last moment he requested to see his wife, who was summoned on the instant. When she appeared at his bedside, he seemed to grow furious at sight of her features, which perhaps brought to mind his first seizure and his first despair. He reproached her with the greatest bitterness for having betrayed him to the police; it must have been her who had given notice of his defalcation at the conscription, as none knew it but herself. He had sent for her but to tell her this, and to bid her not rejoice at his death, as he had given his whole fortune into the keeping of Mons. Sainte —— (whose brother is one of our first *litterateurs*); and although it was her own by right of the marriage contract, he would defy her to draw a farthing from him, were she backed by all the lawyers in Paris! Letters corroborative of this assertion were found in the knapsack, which, in the character of wandering beggar he had adopted for so many years, he had carried on his back, together with the account of his daily expenditure most regularly kept. As he had anticipated, however, Mons. Sainte —— denies all participation in the concealment of the money, and declares the letters found with his signature were but to soothe the irritation of the "*pauvre fou.*"

